# Extracellular Vesicles Isolated from Equine Adipose-Derived Stromal Stem Cells (ASCs) Mitigate Tunicamycin-Induced ER Stress in Equine Corneal Stromal Stem Cells (CSSCs)

**DOI:** 10.3390/cimb46040204

**Published:** 2024-04-09

**Authors:** Justyna M. Meissner, Aleksandra Chmielińska, Ron Ofri, Anna Cisło-Sankowska, Krzysztof Marycz

**Affiliations:** 1 Department of Experimental Biology, Faculty of Biology and Animal Science, Wrocław University of Environmental and Life Sciences, Norwida 27B, 50-375 Wroclaw, Poland; justyna.meissner@upwr.edu.pl; 2 International Institute of Translational Medicine, Jesionowa 11, Malin, 55-114 Wisznia Mala, Poland; 117545@student.upwr.edu.pl (A.C.); okulistavet@gmail.com (A.C.-S.); 3 Koret School of Veterinary Medicine, Hebrew University of Jerusalem, P.O. Box 12, Rehovot 7610001, Israel; ron.ofri@mail.huji.ac.il; 4 Department of Medicine and Epidemiology, School of Veterinary Medicine, University of California, Davis, CA 95516, USA

**Keywords:** ASCs-EVs, corneal stem cells, ER stress, corneal injury, inflammation

## Abstract

Corneal ulcers, characterized by severe inflammation of the cornea, can lead to serious, debilitating complications and may be vision-threatening for horses. In this study, we aimed to investigate the role of endoplasmic reticulum (ER) stress in corneal stem progenitor cell (CSSC) dysfunction and explore the potential of equine adipose-derived stromal stem cell (ASC)-derived extracellular vesicles (EVs) to improve corneal wound healing. We showed that CSSCs expressed high levels of CD44, CD45, and CD90 surface markers, indicating their stemness. Supplementation of the ER-stress-inducer tunicamycin to CSSCs resulted in reduced proliferative and migratory potential, accumulation of endoplasmic reticulum (ER)-stressed cells in the G0/G1 phase of the cell cycle, increased expression of proinflammatory genes, induced oxidative stress and sustained ER stress, and unfolded protein response (UPR). Importantly, treatment with EVs increased the proliferative activity and number of cells in the G2/Mitosis phase, enhanced migratory ability, suppressed the overexpression of proinflammatory cytokines, and upregulated the anti-inflammatory *miRNA-146a-5p*, compared to control and/or ER-stressed cells. Additionally, EVs lowered the expression of ER-stress master regulators and effectors (*PERK*, *IRE1*, *ATF6*, and *XBP1*), increased the number of mitochondria, and reduced the expression of *Fis-1* and *Parkin*, thereby promoting metabolic homeostasis and protecting against apoptosis in equine CSSCs. Our findings demonstrate that MSCs-derived EVs represent an innovative and promising therapeutic strategy for the transfer of bioactive mediators which regulate various cellular and molecular signaling pathways.

## 1. Introduction

Corneal ulceration is a common and painful ocular condition affecting horses, which is characterized by the erosion or breakdown of the cornea, leading to corneal perforation and vision loss. Different hospitals have reported that ulcerative keratitis represents a significant proportion of ophthalmic evaluations, ranging from 24% to 76% [1,2]. The current treatment of corneal ulceration requires the application of local and/or systemic antimicrobials/antifungals, anticollagenases, cycloplegics, and nonsteroidal anti-inflammatory drugs [3,4], and in severe cases, surgery may be necessary [5]. The economic impact of corneal ulceration can be significant for horse owners and racehorse markets, including the cost of treatment, loss of productivity, and reduced performance of pleasure and sport horses [6,7]. The cornea is composed of various cell types, including corneal stromal stem cells (CSSCs), that can differentiate into various corneal cells and are responsible for maintaining and repairing the corneal tissue [8,9,10,11,12,13]. In response to a trauma, a set of immune active cells infiltrate the cornea and release a number of degrading enzymes, proinflammatory cytokines, and immunoglobulins that collectively trigger corneal stromal lysis and necrosis. In this environment, bacteria and fungi can proliferate, contributing to the ulceration and degradation of the cornea [14]. Interestingly, recent studies demonstrated a critical crosstalk between keratocytes and CSSCs activity, which display remarkable expansion capacity, regeneration, and immunomodulatory potential, as well as an ability to differentiate and specialize into mature, functional keratocytes [15,16].

Mesenchymal stem cells (MSCs) have garnered significant interest due to their potential in regenerative medicine [17]. There is growing interest in using corneal stromal stem cells (CSSCs), a type of MSC found specifically in the corneal stroma, regarding the treatment of various corneal diseases and conditions such as corneal ulceration, keratoconus, and corneal scarring [9,10,18,19,20]. Indeed, stem-cell-based therapies for the cornea have been widely regarded as a significant breakthrough due to their potential to replace conventional corneal transplantation methods [20,21,22,23,24,25,26]. Despite the potential benefits of MSC transplantation, there are risks associated with this modality, such as ethical concerns, poor engraftment, reduced post-transplantation viability, and immune responses. Consequently, recent research suggests that MSC-derived extracellular vesicles (EVs) could be a viable alternative. EVs are small membrane-bound particles secreted by cells that contain bioactive molecules such as proteins, lipids, and nucleic acids (including coding RNAs and different types of noncoding RNAs [27]). MSC-derived EVs have shown similar therapeutic potential to MSCs in various tissue regeneration applications and are emerging as a promising treatment strategy for corneal diseases. MSC-EVs may have significant advantages over MSCs, such as improved safety profiles and ease of administration, making them an appealing option for the development of novel corneal regeneration therapeutics [28,29]. EVs, which are naturally released from cells, are a diverse group of signaling molecules that are enclosed within a lipid bilayer membrane. Many growth factors have been identified in EVs, all of which play a pivotal role in the restoration of physiological functions in injured tissues [30]. EVs have been implicated in the regulation of inflammation across a large plethora of diseases, including cancer [31], and autoimmune [32,33], cardiovascular [34], neurodegenerative [35], infectious diseases [36] and corneal fibrosis [37]. The immunomodulatory effect of EVs has been proposed as one of the main mechanisms supporting corneal wound healing [37,38,39,40,41]. Indeed, early investigations into EVs following keratectomy (a corneal excision procedure) provided some evidence of their release into the corneal stroma [42]. Subsequent studies examined the impact of EVs on corneal neovascularization and revealed that the corneal epithelium releases EVs in response to injury [39,43]. Other studies have shown that limbal stromal-cell-derived EVs may influence the proliferation and migration of limbal epithelial stem cells in vitro, with different outcomes depending on the cell source [44]. Further in vivo research has shown that mesenchymal stem-cell-derived EVs can promote improved corneal epithelial wound healing and scarless stromal recovery post-injury [40,45]. This regulation is attained by targeting proinflammatory cytokines, including interleukin-1 (IL-1), TGF-β, tumor necrosis factor-alpha (TNF-α), and IL-6, as well as inflammation-related miRNAs, including miR-155 and miR-146a, which are the key regulators in corneal wound healing. However, there is limited understanding of the impact of EVs on endoplasmic reticulum (ER) stress attenuation [40,45,46,47].

ER stress and the unfolded protein response (UPR) are involved in the pathogenesis of several ocular diseases [48], including cataract [49], retinitis pigmentosa [50], glaucoma [51], and corneal scarring in patients with diabetic ocular diseases [52,53]. The accumulation of misfolded proteins leads to inflammation [54], fibrosis, and apoptosis [54,55,56], which contribute to disease progression. To reduce ER stress and the accumulation of toxic misfolded proteins, cells activate the UPR, which rescues them from an unfavorable environment [57]. The UPR is mediated by three major ER transmembrane receptors: activating transcription factor 6 (ATF6), protein kinase R(PKR)-like endoplasmic reticulum kinase (PERK), and inositol requiring enzyme 1 (IRE1) [58]. Under normal physiological conditions, these effectors are inactive due to binding to the intraluminal ER chaperone protein GRP78 [59]. In response to ER stress, other UPR markers, such as phosphorylated α subunit of eukaryotic initiation factor 2 (phospho-eIF2α) and C/EBP homologous protein (CHOP), are also recruited [60]. Failure to alleviate ER stress through the UPR can trigger pathological processes including inflammation, oxidative stress, fibrosis, and apoptosis [54,61,62]. In corneal diseases, sustained ER stress has been found to trigger corneal stromal cells apoptosis, excessive senescence, and reduced migratory capacity, thereby reducing their regenerative and repair potential within the injured cornea, resulting in corneal opacity [63]. Therefore, targeting ER stress may effectively reduce inflammation, fibrosis, and scarring resulting from corneal ulceration.

In this study, our aim was to investigate the therapeutic effects of EVs secreted from equine adipose-derived stem cells on equine CSSCs regeneration, using a tunicamycin-induced ER stress model [64]. Our findings demonstrate that EVs can improve the survival, proliferation, and expansion of CSSCs under ER stress condition by inhibiting apoptosis and modulating inflammation and fibrosis of corneal injury.

## 2. Materials and Methods 

All reagents used in this study were purchased from Sigma Aldrich (Carlsbad, CA, USA), unless indicated otherwise.

### 2.1. Experimental Model and Subject Details

All the equine cornea samples were collected from animal slaughter with the agreement of the owners. Equine adipocyte stem cells were acquired from the biobank collection of the Department of Experimental Biology, University of Environmental and Life Sciences, Wroclaw, Poland.

### 2.2. Cornea Tissue Harvesting and CSSC Isolation

Equine cornea was collected from a 3-month-old foal cadaver donor with the consent of the owner. All steps involved in tissue isolation were performed under strictly sterile conditions using an enzymatic–mechanical method. Briefly, the cornea was washed twice in sterile phosphate-buffered saline (PBS) containing a 1% penicillin–streptomycin (PS) antibiotic mixture (Biowest, Bradenton, FL, USA). Subsequently, corneal limbal tissue was separated and cleared from the iris, epithelium, and endothelium, and cells were isolated using the protocol described by Nagymihály et al. [65]. The isolated corneal stromal stem cells were subsequently cultured in Dulbecco’s Modified Eagle’s Medium (DMEM) containing 1 g/L glucose, supplemented with 10% fetal bovine serum (FBS) and 1% penicillin–streptomycin antibiotic solution. Cells were grown in an incubator with 5% CO_2_ and 95% humidity at 37 °C. The cells were passaged 3 times and then used for further studies.

### 2.3. Phenotypic Characterization of Corneal Stromal Stem Cells

The phenotyping of the isolated corneal stromal stem cells was conducted by analysis of expression of surface markers, including CD44, CD45, CD90, and CD200, using Becton Dickinson FACS Calibur Flow Cytometer. In brief, cells were harvested with Trypsin-EDTA 1× in PBS (Biowest, Bradenton, FL, USA), washed with PBS, and suspended at a final density of 5 × 10^5^ cells/mL. The obtained cells were subsequently labeled with specific antibodies (anti-CD45; Novus Biologicals, Littleton, CO, USA, NB1006590APC, anti-CD44; R&D Systems, Minneapolis, MN, USA, MAB5449, anti-CD90, ab225; Abcam, Cambridge, UK, anti-CD200, MBS3216028, My BioSource, San Diego, CA, USA) and incubated in the dark at room temperature for 30 min. All samples were then analyzed using FCS Express7.

### 2.4. ASC-EVs Isolation and Characterization

Equine ASCs were obtained from the collection of the Department of Experimental Biology, University of Environmental and Life Sciences, Wroclaw, Poland. Cells were grown in an incubator with 5% CO_2_ and 95% humidity at 37 °C. The cells were passaged 3 times and then used to produce EVs. EVs were prepared according to the method previously described by Szatanek R. et al. [66]. Briefly, conditioned culture media were collected from healthy horses’ ASCs after 3 days culturing in FBS-free DMEM containing 1 g/L glucose, supplemented with 1% PS for 24 h. EVs were subsequently isolated by centrifuging the collected media at 300× *g* for 10 min, 2000× *g* for 10 min, and 100,000× *g* for 30 min at 4 °C. The obtained pellet was washed several times using PBS. The obtained EVs were suspended in PBS and used in subsequent stages of the study. EVs were characterized by staining the marker CD63 by flow cytometry as previously described [67]. The EVs obtained from ASCs medium were quantified with the Pierce™ BCA Protein Assay Kit (Thermo Fisher Scientific, New York, NY, USA).

### 2.5. Experimental Model Setting

CSSCs were seeded onto a 24-well plate at a density of 2.5 × 10^4^ cells per well, and left to attach for 24 h. Culture media of the experimental groups were afterwards changed to a medium containing 10 mmol/mL tunicamycin to induce ER stress. After 24 h, the medium was changed in the EVs-treated group to a medium containing EVs in a final concentration of 30 μg/mL. Thereafter, the control group medium was the same as the medium in which the corneal cells were cultured. After 24 h, the cells were collected for further testing.

### 2.6. Proliferation Assay

All proliferation-related procedures were performed after 24 h treatment of cells with EVs and/or with tunicamycin. The viability rate was assessed with a resazurin-based test (TOX8 In Vitro Toxicology Assay Kit, Sigma Aldrich, Poznan, Poland). To perform the test, culture media were replaced with a 10% resazurin solution prepared in complete culture medium. The cells were then incubated at 37 °C for 2 h. Then, the post-culture media were placed in a 96-well plate and spectrophotometric measurement was performed at 600 nm and 690 nm as reference wavelengths. The results were analyzed with GraphPad Prism 8 Software.

For the clonogenic assay, cells were seeded in a 6-well plate at an initial density of 100 cells per well. After overnight incubation for cellular attachment, groups were treated with either tunicamycin and/or EVs (30 μg/mL). After 7 days, cells were fixed in 4% paraformaldehyde (PFA) (Sigma-Aldrich, P6148) and stained for 5 min at room temperature with pararosaniline solution. Colonies containing more than 50 cells were counted, and colony-forming unit (CFU) rate was obtained using the formula described by Kornicka K. et al. [68].

A scratch test was also performed to evaluate cell migration capacity. For this purpose, the cells received respective treatments as described above prior to cells monolayers scratching. A series of photomicrographs were taken at 0 h, 6 h, 24 h, and 48 h, and cell migration parameters were analyzed using GraphPad Prism 8 Software.

### 2.7. Visualization of Cells’ Mitochondria and Cytoskeleton

Treated and untreated CSSCs morphology was assessed by confocal microscopy. MitoRed dye (1:1000 in medium) was added to the culture medium of cells after each related treatment and incubated for 30 min at 37 °C to stain the mitochondrial network; excess MitoRed was then discarded and washed with PBS. Cells were fixed in 4% PFA for 40 min at room temperature in the dark. For cytoskeleton visualization, all cell groups were washed with PBS, fixed in 4% PFA for 40 min at room temperature, and permeabilized in a 0.1% Triton X-100 solution for 15 min. Actin filaments were then stained using atto-488-labeled phalloidin (1:800 in PBS) for 40 min, in the dark at room temperature. The nuclei were counterstained with DAPI (Faramount Aq Mounting Medium, Agilent, Poznan, Poland). The cells were observed and imaged using a confocal microscope (Observer Z1 Confocal Spinning Disc V.2 Zeiss, Poznan, Poland).

### 2.8. Immunofluorescence Staining

Healthy control, ER-stressed cells, and ER-stressed cells treated with EVs were fixed with 4% PFA for 20 min and then permeabilized in a 0.1% Triton X-100 solution for 15 min at room temperature. Cells were then incubated overnight with the primary antibodies (Table 1) diluted in 10% Normal Goat Serum in PBS. Next, the cells were incubated with the Rb/Ms Atto-594 secondary antibody diluted in PBS (1:1000, Sigma). Cell nuclei were stained with DAPI (Faramount Aq Mounting Medium, Agilent, Poznan, Poland), and photographed with a confocal microscope (Observer Z1 Confocal Spinning Disc V.2 Zeiss, Poznan, Poland).

### 2.9. mRNA Expression Analysis

Total RNA was isolated with the EXTRAzol reagent (Blirt, Gdańsk, Poland) in accordance with the manufacturer’s instructions. The concentration, as well as quality and purity, of isolated RNA were verified with a nanospectrophotometer (Epoch, BioTek, Winooski, VT, USA). Transcription of RNA into cDNA was performed using the Takara PrimeScriptTM RT Reagent Kit with gDNA Eraser (Perfect Real Time). Real-time polymerase chain reaction (qPCR) was performed using the SensiFast SYBR & Fluorescein Kit (Bioline, London, UK) and a CFX ConnectTM Real-Time PCR Detection System (Bio-Rad, Hercules, CA, USA). Each reaction amplified 150 ng of cDNA in a final volume of 10 µL. The thermal cycling conditions were as follows: 95 °C for 2 min followed by 41 cycles at 95 °C for 15 s, annealing for 30 s and elongation at 72 °C for 15 s. The RT-qPCR reaction was repeated at least three times. The relative expression levels of each targeted gene (Table 2 and Table 3) were normalized in relation to the expression of the glyceraldehyde 3-phosphate dehydrogenase (GAPDH) using the 2^−ΔΔCq^ method.

In order to conduct RT-qPCR for miRNA, the Mir-X miRNA First Strand Synthesis Kit (Takara, Kusatsu, Japan) was used. Briefly, gDNA traces were removed by treating the RNA with the DNase I, RNase-free in 10× reaction buffer with MgCl2 and water, at 37 °C for 30 min. The proper volume of obtained RNA was mixed with mRQBuffer (2x and mRQEnzyme. The reaction mixture was incubated at 37 °C for 1 h, then at 85 °C for 5 min. The expression level of miRNA was analyzed by real-time PCR using the MicroRNA first-strand synthesis kit according to the instructions provided by the manufacturer. Briefly, the reaction mixture contained water, SensiFast SYBR & Fluorescein Kit (Bioline, London, UK), miRNA-specific primer (Table 3), mRQ 3’primer, and cDNA. As a reference sample, U6F primer and U6R primer were used. The relative expression level was calculated by comparison of the tested groups with the control group using the 2^−ΔΔCq^ method.

### 2.10. Oxidative Stress Analysis

Intracellular oxidative stress factors, including reactive oxygen species (ROS), were determined using a commercial kit (Muse^®^ Oxidative Stress kit). The procedure followed the protocol provided by the manufacturer. Cells were resuspended in 1× Assay Buffer, and approximately 1 × 10^6^ cells/mL were obtained. A working solution was then prepared containing the Muse^®^ Oxidative Stress intermediate solution (1:80). Cells were added to the prepared solution and incubated at 37 °C for 30 min. Results were acquired using a Muse™ Cell Analyzer (Merck, Darmstadt, Germany).

### 2.11. Cell Cycle Analysis

Cell cycle analysis was performed using the Muse™ Cell Cycle kit [69,70,71]. As recommended by the manufacturer, treated and untreated cells were suspended in ice-cold 70% ethanol and incubated at −20 °C overnight. The cells were then centrifuged and resuspended in 1× PBS. The Muse Cell Cycle Reagent was added to the cells which were then incubated for 30 min at room temperature in the dark. Distribution of cells across the four cell cycle phases was established with the Muse™ Cell Analyzer (Merck, Germany).

### 2.12. Apoptosis Assay

Cell viability and apoptosis were determined using the Muse^®^ Annexin V & Dead Cell Kit. Treated and untreated cells were prepared in accordance with the manufacturer’s instructions. Muse^®^ Annexin V & Dead Cell reagent was added to the cells. They were then incubated in the dark for 20 min at room temperature. Analysis of apoptotic cells population was performed with a Muse Cell Analyzer (Merck, Germany).

### 2.13. Senescence β-Galactosidase Staining

Detection of the aging-related lysosomal enzyme (SA-β-Gal) was performed with a Senescence Cells Histochemical Staining Kit in accordance with the manufacturer’s instructions. Briefly, cells were fixed by incubation for 6 min with 1× Fixation Buffer diluted in PBS. The buffer was changed to Staining Mixture, containing Staining Solution 10×, Reagent B, Reagent C, X-gal Solution, and ultrapure water. Cells were then incubated at 37 °C overnight, and subsequently examined using an inverted microscope (Leica, Wetzlar, Germany). 

### 2.14. Statistical Analysis

Results were analyzed by one-way variance analysis (ANOVA) using GraphPad Software 8 (San Diego, CA, USA) according to Tukey’s post hoc test. Statistically significant results (comparison of cells treated with EVs to cells with ER stress) were marked with an asterisk, respectively, for *p* < 0.05 (*), *p* < 0.01 (**), and *p* < 0.001 (***). Statistically significant results (comparison of untreated cells control to cells treated with tunicamycin (ER_s) and comparison of EVs to ASCs) were marked with a hashtag, respectively, for *p* < 0.05 (#), *p* < 0.01 (##) and *p* < 0.001 (###). Results are presented as mean ± SD from at least three independent experiments.

## 3. Results

### 3.1. EVs Characterization

**Figure 1:**
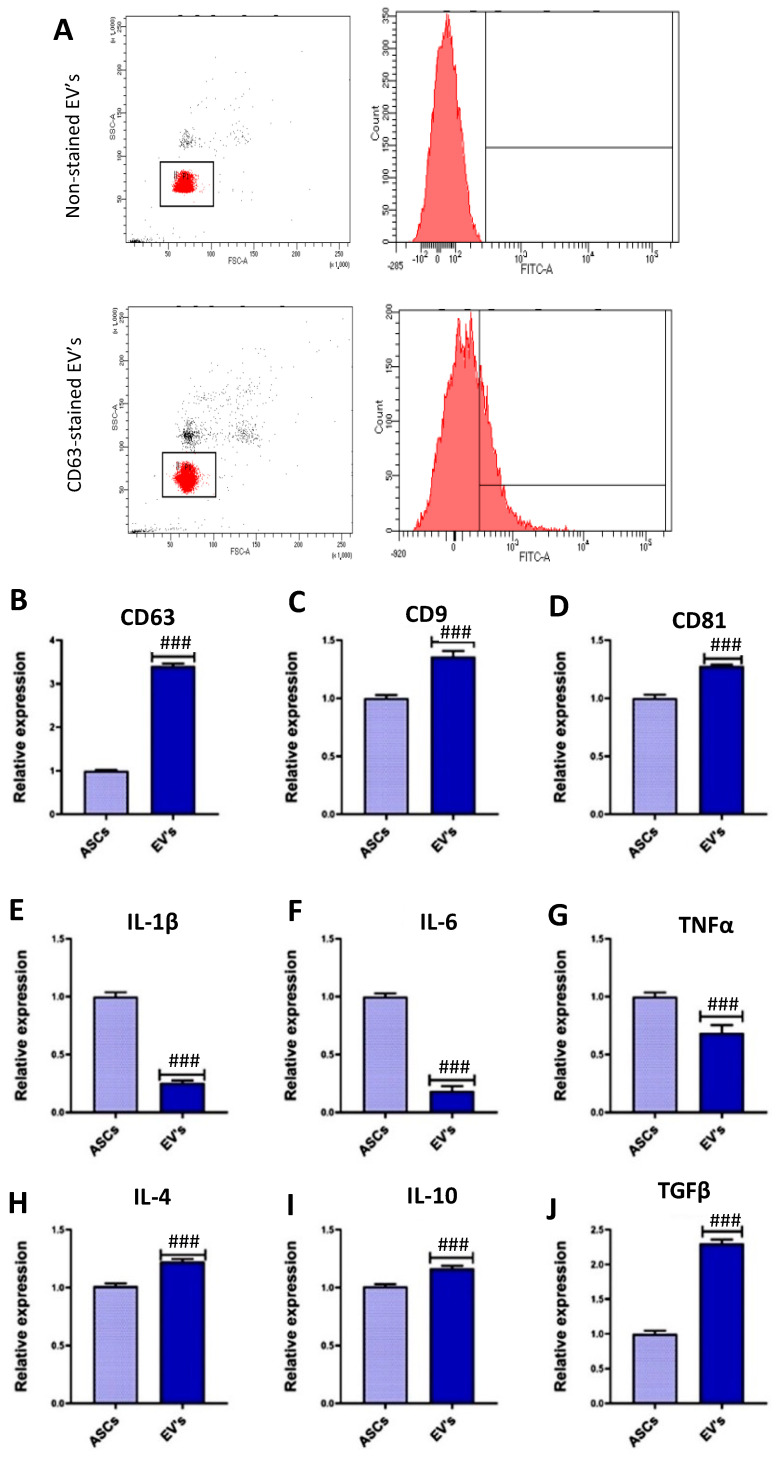
Characterization of equine ASC-derived EVs. (**A**) Isolated EVs were stained with an anti-CD63 antibody and visualized by flow cytometry. Red dots correspond to the gated population. (**B**–**D**) Relative gene expression analysis by qRT-PCR of surface markers (*CD63*, *CD9*, *CD81*) in EVs and ASCs. (**E**–**J**) Relative gene expression analysis by qRT-PCR of inflammation-related genes (*IL-1β*, *IL-6*, *TNFα*, *IL-4*, *IL-10*, *TGFβ*) in EVs and ASCs. Values are presented as mean ± SD. In all nine markers (**B**–**J**), the difference between the groups was significant compared to ASCs (### *p* < 0.001).

Recently it has been demonstrated that EVs are characterized by CD63, CD9, and CD81 surface markers [72]. The characterization of the isolated EVs was performed by flow cytometry analysis, staining one of the EVs surface markers, CD63 [67]. In this regard, we observed that the isolated EVs population was CD63-positive (Figure 1A). Then, we quantified the mRNA levels of these surface markers using RT-qPCR, as described in other studies [27,73,74]. Compared to ASCs, the equine ASC-derived EVs displayed increased mRNA levels of *CD63*, *CD9*, and *CD81* (Figure 1B–D). Moreover, the mRNAs of some inflammation modulators were also studied in the EVs by RT-qPCR, showing a reduction in the mRNA levels of several proinflammatory genes compared to ASCs, including *IL-1β*, *IL-6*, and *TNFα* (Figure 1E–G). Importantly, we detected increased mRNA levels of several anti-inflammatory mediators in the ASC-derived EVs compared to ASCs, including *IL-4*, *IL-10*, and *TGFβ* (Figure 1H–J), suggesting that these EVs might have a modulatory role in inflammation processes. The increased levels of mRNA found in the EVs compared to ASCs are in accordance with the fact that RNA contained in EVs is not exposed to the cellular RNases and, therefore, have a longer lifespan [75].

### 3.2. Effect of EVs on CSSCs Cells’ Stemness

**Figure 2:**
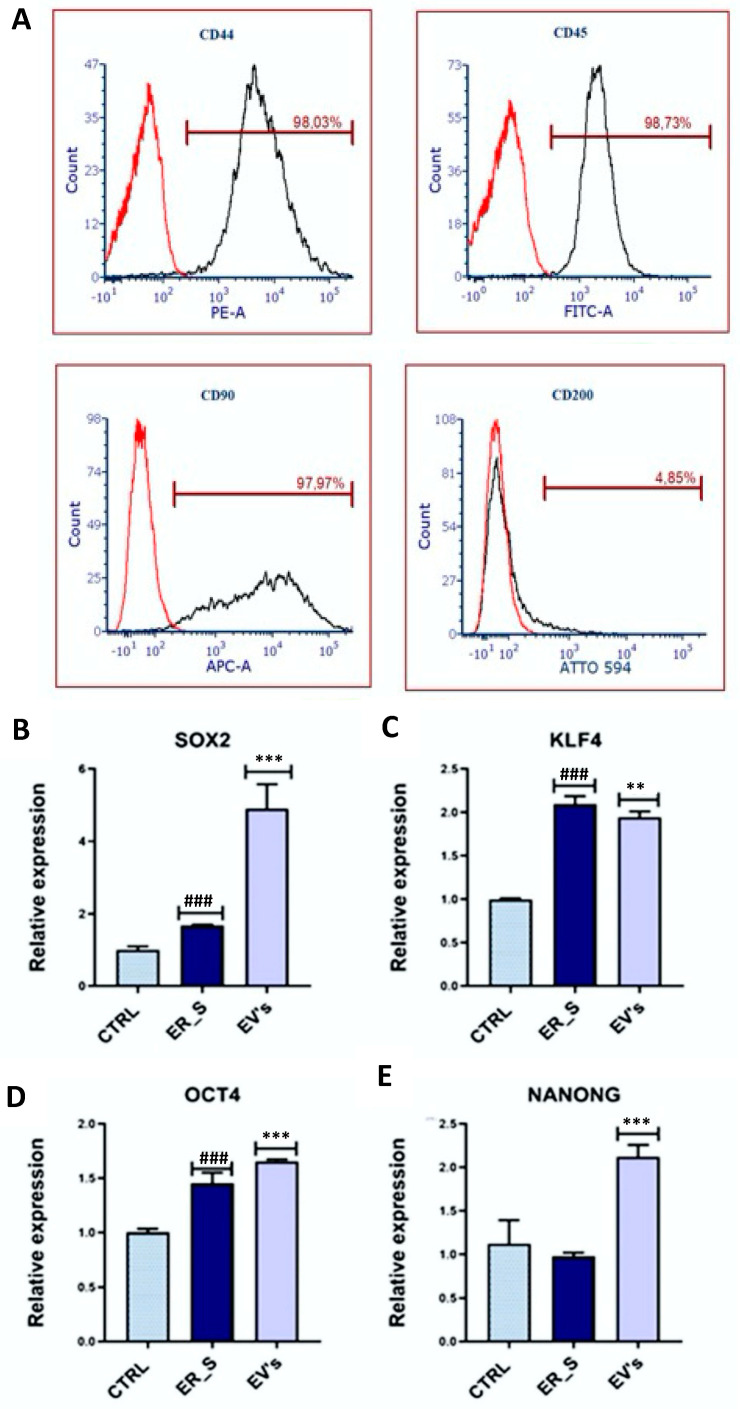
Effects of EVs on CSSCs stemness. (**A**) Analysis of surface markers in isolated CSSCs by flow cytometry. Red line refers to non-stained control population; black line refers to the stained population. (**B**–**E**) Analysis of the relative expression of *SOX2*, *KLF4*, *OCT4*, and *NANOG* by RT-qPCR. Values are expressed as mean ± SD. ** *p* < 0.01, ^###^ or *** *p* < 0.001. Statistical significance indicated as asterisks (*) when comparing the EVs group to ER_s (ER stress) group and as number signs (#) when comparing ER_s group to control (CTRL).

Isolated CSSCs were characterized by immunophenotyping of key mesenchymal stem cells surface markers. Flow cytometry analysis showed that equine CSSCs presented a high expression of the surface markers CD44 (98.03%), CD45 (98.73%), and CD90 (97.97%) and low expression of CD200 (4.85%) (Figure 2A), confirming their mesenchymal origin. Then, the effect of EVs on cells’ stemness under ER stress conditions was evaluated by measuring by qRT-PCR the expression of the markers *SOX2*, *KLF4*, *OCT4*, and *NANOG*, known to be expressed in CSSCs [18,76,77]. For RT-qPCR, first, CSSCs were treated with tunicamycin to induce ER stress, increasing the gene expression levels of *SOX2*, *KLF4*, and *OCT4* (Figure 2B–D). Interestingly, EVs conditioning increased the expression of the *SOX2*, *OCT4*, and *NANONG* genes compared to the cells treated with tunicamycin (Figure 2B,D,E), although *KLF4* expression in EVs was reduced (Figure 2C). These results suggest that ASC-derived EVs have the potential to induce the cell stemness in CSSCs.

### 3.3. EVs Promote the Proliferation of Corneal Stromal Stem Cells and Protect from Apoptosis

**Figure 3:**
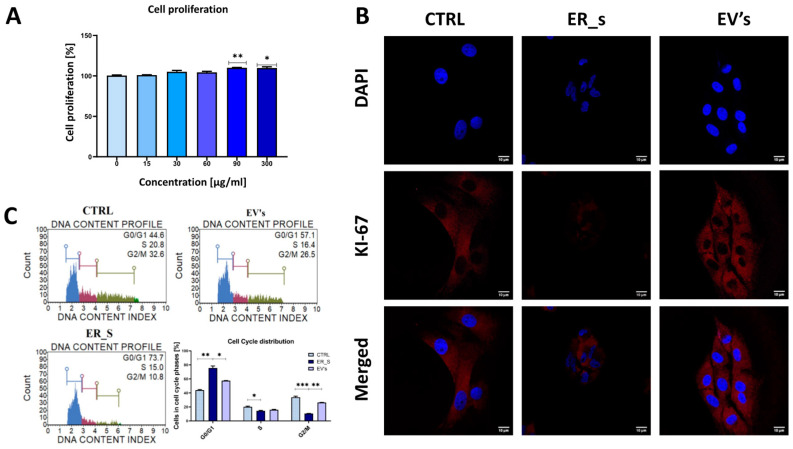
EVs promote the proliferation of corneal stromal stem cells. (**A**) The proliferation rate of CSSC treated with EVs is shown by TOX8 assay. The nontreated cells are normalized to a 100% proliferation. (**B**) Immunostaining of control (CTRL), ER_s, and EVs cells for Ki-67 using specific Atto 594 antibodies (red), while the nuclei were stained with DAPI (blue). Bar 10 μm. (**C**) Evaluation of cell cycle with Muse Analyzer Flow Cytometry for control, ER_s, and EVs groups. All values expressed as mean ± SD. * *p* < 0.05, ** *p* < 0.01, *** *p* < 0.001.

EVs are known to contain a number of growth factors and other cellular proliferation and survival mediators. Therefore, we evaluated the potential proliferative capacity of EVs on corneal stromal stem cells. As expected, none of the tested concentrations of isolated EVs had adverse effects on CSSCs viability (Figure 3A). Most importantly, EVs concentrations of 90 and 300 μg/mL significantly increased the proliferative capacity of CSSCs, compared to the nontreated control (Figure 3A), indicating a likely proliferative effect of the tested EVs on stem cells. Exposure of CSSCs to tunicamycin decreased the fluorescent signal of the Ki-67 nuclear proliferation protein, while EV treatment restored the control levels of Ki-67 (Figure 3B), restoring the proliferative and metabolic activity of CSSCs following ER stress induction. The cell distribution across the various cell cycle phases depends on the cell type, cell size, and the initial DNA content. The MUSE Analyzer automatically defines the cell cycle phase based on the cell population’s gating, which depends on fluorescence intensity and cell size [69,70,71]. In this regard, tunicamycin increased the accumulation of ER-stressed cells in the G0/G1 phase of the cell cycle, and decreased the number of cells in the G2/Mitosis phase, compared to control (Figure 3C). Interestingly, treatment with EVs decreased the number of cells engaged in the G0/G1 phase and increased the number of cells engaged in G2/Mitosis phase of the cell cycle, reestablishing the phenotype observed in the nontreated control (Figure 3C).

**Figure 4:**
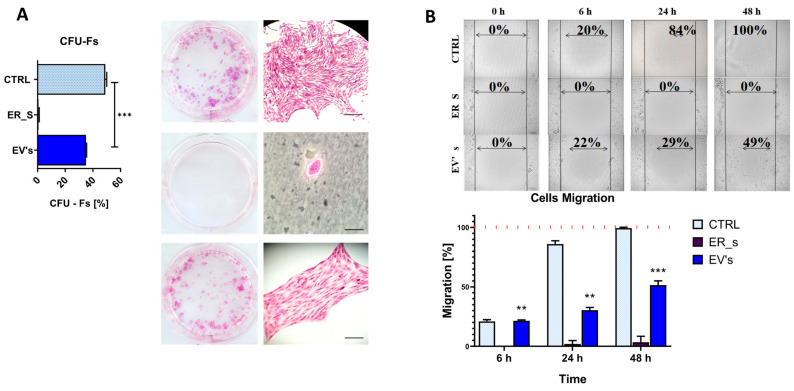
EVs promote the proliferation of CSSC. (**A**) Cell colony formation under different condition (CTRL, nontreated control; ER_S, ER-stress control; EVs-treated, cells treated with EVs) is shown in a clonogenic assay. Pictures of the wells, as well as pictures of the colonies under the microscope, are shown. The diagram shows the percentage of cells’ ability to form colonies in the three groups. Bar in top and bottom images, 30 μm; Bar in middle image, 15 μm. (**B**) Scratch assay. Visualization of cells under different conditions at 0 h, 6 h, 24 h, and 48 h after scarring. The black vertical lines show the edges of the scar, while the arrow lines show the confluence of the scar. The diagram shows the percentage of cell migration after 6 h, 24 h, and 48 h. All values expressed as mean ± SD. ** *p* < 0.01, *** *p* < 0.001.

To further support these results, a clonogenic assay was conducted. Supplementation of tunicamycin to CSSCs inhibited the ability to form colonies (Figure 4A); however, treatment with EVs at 30 μg/mL partially restored the control phenotype (Figure 4A). The migratory ability was then evaluated by the scratch assay, showing that ER-stressed cells presented an expansion rate of 0% (Figure 4B). In this regard, we ruled out the possibility that cells were not viable upon treatment with tunicamycin, given the active gene expression profile of this condition detailed in this study, and that the used tunicamycin concentration is within the range of other studies where cells are subjected to ER stress [78]. Most importantly, treatment with EVs reestablished the expansion rate up to a 49%, closer to that observed in the nontreated control at 48 h post-scratch induction (Figure 4B).

**Figure 5:**
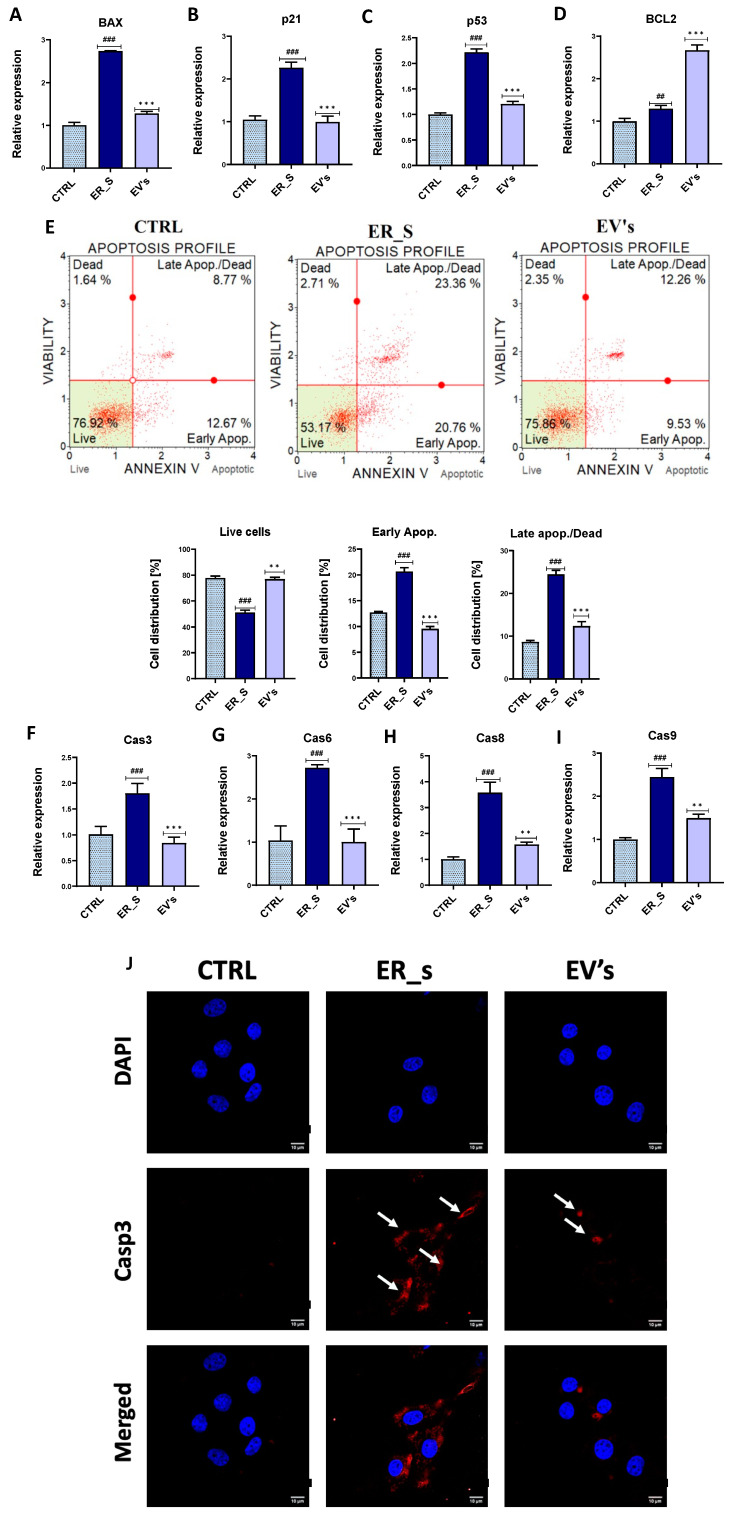
EVs protect from apoptosis. (**A**–**D**,**F**–**I**) Visualization of the relative expression of major genes during apoptosis. (**E**) Evaluation of early and late apoptosis by the Muse Analyzer Flow Cytometry. The percentage of living cells, and cells in different stages of apoptosis, is presented in the histograms. (**J**) Immunostaining of control (CTRL), ER_s, and EVs cells with Cas3 antibody (red), nuclei stained with DPAI (blue). Arrows indicate signal of Cas3 antibody. Bar 10 µm. All values expressed as mean ± SD. ^##^ or ** *p* < 0.01, ^###^ or *** *p* < 0.001. Statistical significance indicated as asterisks (*) when comparing the EVs group to ER_s group and as number signs (#) when comparing ER_s group to control.

To evaluate the antiapoptotic effect of the EVs, selected apoptosis-related markers were analyzed by qRT-PCR under the ER stress conditions induced by tunicamycin. Tunicamycin increased the expression of the proapoptotic genes *BAX*, *p21*, and *p53* (Figure 5A–C), while supplementation with EVs decreased the expression of these proapoptotic master regulators (Figure 5A–C) and increased the expression of the apoptosis suppressor *BCL-2* (Figure 5D). We observed that control cells had a basal degree of apoptosis activation, which could result from the stress of these primary cells when cultured in in vitro conditions (Figure 5E). Importantly, the percentage of apoptotic cells increased in the ER-stressed conditions (Figure 5E), while treatment with EVs showed an increased number of viable cells and a reduced number of cells undergoing apoptosis at both early and late stages, compared to ER-stressed cells (Figure 5E). Likewise, tunicamycin triggered the induction of the gene expression of the main caspases involved in apoptosis, including *Cas3*, *Cas6*, *Cas8*, and *Cas9* (Figure 5F–I). Accordingly, increased expression of Caspase 3 protein was observed in the immunofluorescence microscope analysis in CSSCs (Figure 5J). Most importantly, treatment with EVs decreased the relative expression of the same caspases’ transcripts (caspases 3, 6, 8, and 9) (Figure 5F–I), and reduced the ER-stress-induced Caspase 3 protein expression (Figure 5J). It is noteworthy that in control cells we detected around 10% of annexin V-positive cells, but no caspase 3 staining in the cytosol (Figure 5E,J). This might be due to the timing of the events during the apoptotic pathway, where a basal level of apoptosis was induced, and that the remaining levels of caspase 3 in the cytosol were nondetectable, while the phosphatidylserine was still detectable by the annexin V flow cytometry staining (Figure 5E,J). Altogether, these results suggest that EVs reverse tunicamycin-induced apoptosis and enhance overall cellular viability, proliferation, and migratory potential.

**Figure 6:**
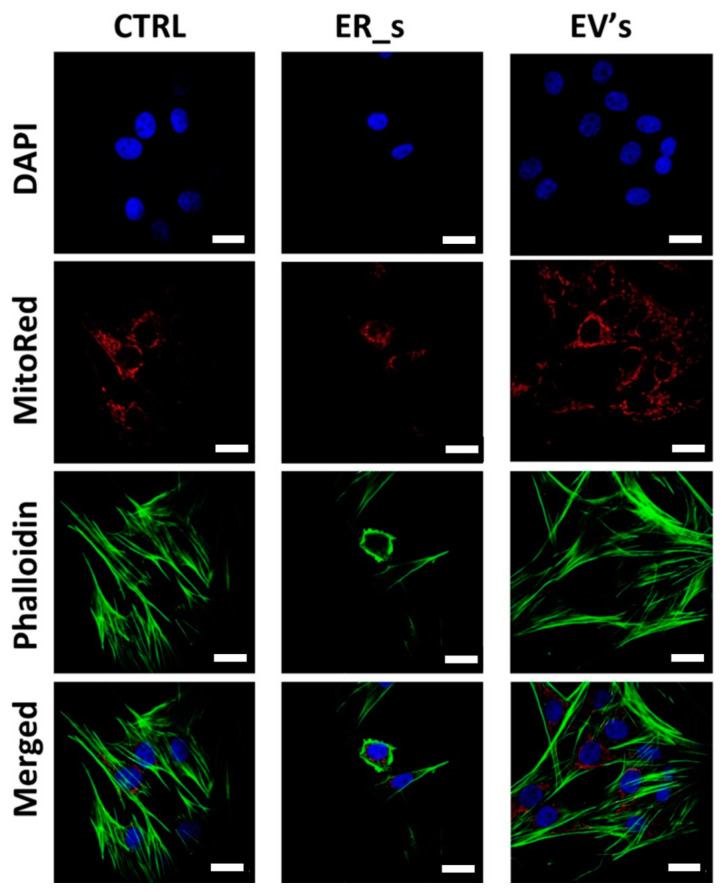
EVs protects from ER-stress-induced cytoskeleton rearrangement. Morphological changes for treated and untreated cells were estimated with confocal microscopy. Cells were stained with DAPI (nuclei, blue), phalloidin (F-actin, green), and MitoRed dye (mitochondria, red). Bar 10 μm.

It is well documented that the ER function is related to the structure of the cytoskeleton, and that ER stress is associated with impairment and structural alterations of mitochondria [79,80,81,82]. In this line of thought, we aimed to evaluate how the tunicamycin-induced ER stress altered the architecture of the cytoskeleton and mitochondrial network in CSSCs, together with the ability of EVs to restore the nontreated condition phenotype (Figure 6). The use of tunicamycin to induce ER stress in CSSCs reduced cell cytoskeleton, as well as the mitochondrial network, compared to the control group (Figure 6). Interestingly, EVs-treated cells were characterized by improved actin quantity, wide distribution, and the number of mitochondria, improving the appearance of the cytoskeleton and nucleus compared to that of the ER_s group (Figure 6). Altogether, these findings demonstrate that EVs promote cellular proliferation in CSSCs and regulate the programmed apoptosis cell death pathway to promote cell survival.

### 3.4. EVs Mitigate Inflammation in CSSCs

**Figure 7:**
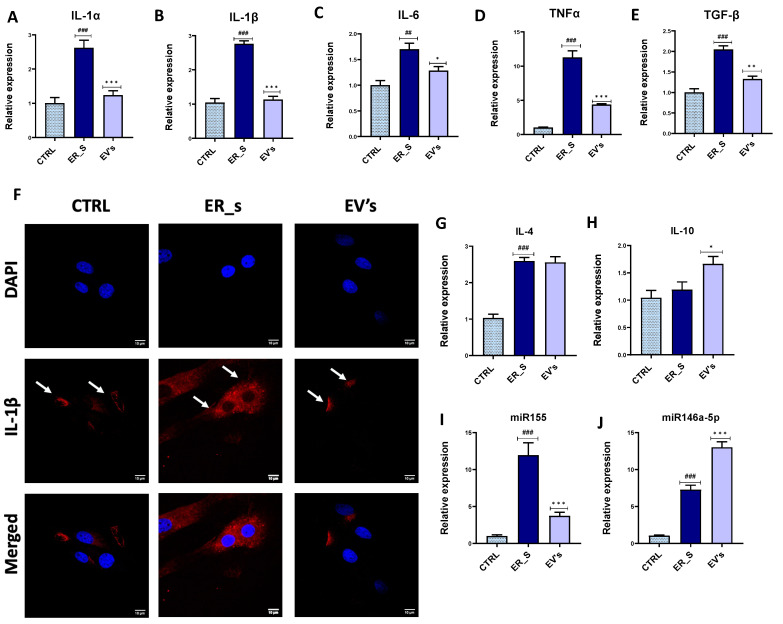
EVs mitigate inflammation in CSSCs. (**A**–**E**,**G**–**J**) Analysis of the relative expression of the proinflammatory and anti-inflammatory transcripts by RT-qPCR. (**F**) Immunostaining of control, ER_s, and EVs-treated cells with IL-1β antibody(red) and nuclei stained with DAPI (blue), bar 10 µm. Arrows indicate the presence of IL-1β antibody signal. Values expressed as mean ± SD. * *p* < 0.05, ^##^ or ** *p* < 0.01, ^###^ or *** *p* < 0.001. Statistical significance indicated as asterisks (*) when comparing the EVs group to ER_s group and as number signs (#) when comparing ER_s group to control.

EVs have been previously characterized by their anti-inflammatory and immunomodulatory features. Our results show that tunicamycin induced inflammation in CSSCs by increasing the expression of proinflammatory genes, including *IL-1α*, *IL-1β*, *IL-6*, *TNF-α*, and profibrotic *TGF-β* (Figure 7A–E). Treatment of CSSCs with EVs significantly reduced the expression of the *IL-1α*, *IL-1β*, *IL-6*, *TNF-α*, and *TGF-β* genes during the ER-stressed cells (Figure 7A–E). Furthermore, the expression of *IL-1β* in CSSC was visualized by confocal microscopy, where a strong fluorescent signal for intracellular IL-1β was detected in ER-stressed cells (Figure 7F), compared to the signal in control cells. Interestingly, the levels of cellular IL-1β during ER stress decreased upon treatment with EVs (Figure 7F). Importantly, the expression of the anti-inflammatory IL-4 gene (Figure 7G) appeared to be higher in the ER-stressed group levels than in the nonstressed control group, while expression of IL-10 remained unchanged (Figure 7H). In this regard, EVs supplementation did not affect the expression of IL-4 (Figure 7G) but increased the expression of IL-10 compared to tunicamycin-treated cells, suggesting that EVs may potentiate the immunomodulatory properties of CSSCs (Figure 7H). Moreover, our results demonstrate that, compared to the ER-stressed condition, EVs-treated cells exhibited downregulated expression of the proinflammatory molecule miR155 and higher expression levels of the anti-inflammatory 146a-5p miRNA (Figure 7I,J).

**Figure 8:**
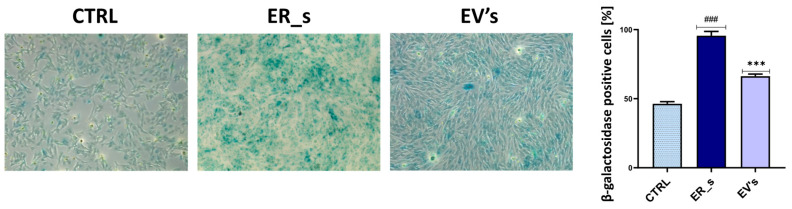
EVs reduce senescence in CSSCs. Cellular senescence was measured using the SA-β-galactosidase kit. The percentage of β-galactosidase-positive cells is presented. Values expressed as mean ± SD. ^###^ or *** *p* < 0.001. Statistical significance indicated as asterisks (*) when comparing the EVs group to ER_s group and as number signs (#) when comparing ER_s group to control.

Because premature senescence is considered as a hallmark of stem cell exhaustion, increased inflammation, and overall cellular stress, we tested the ability of EVs to reverse ER stress induced by β-galactosidase overactivation. As shown in Figure 8, CSSCs exposed to tunicamycin were characterized by a marked increase in the number of β-galactosidase-positive cells compared to control cells. Interestingly, treatment with EVs reduced the prevalence of the premature senescence-like phenotype, decreasing β-galactosidase-positive cells from 100% to 75% in the treated and nontreated tunicamycin-challenged groups, respectively (Figure 8). This suggests that treatment with EVs reversed the accumulation of cellular stress signals which initiate senescence-like events. Collectively, these results show that EVs regulate the cellular inflammatory process by modulating the expression of both pro- and anti-inflammatory mediators in CSSCs.

### 3.5. EVs Reduce ER Stress in CSSCs

**Figure 9:**
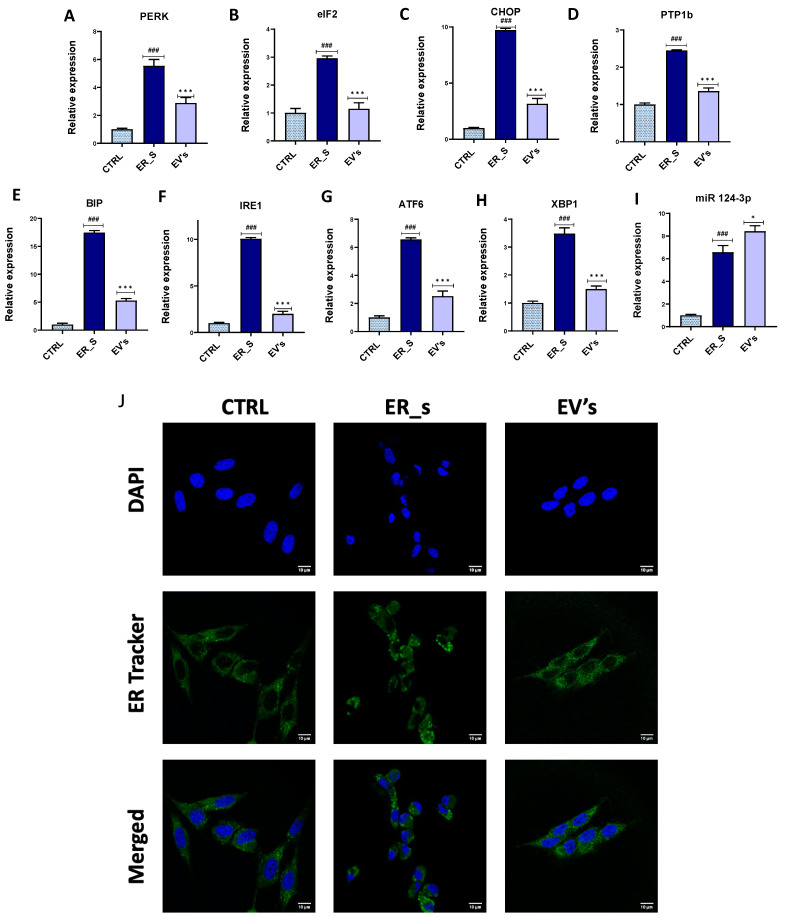
EVs reduce ER stress in CSSCs. (**A**–**I**) The mRNA levels of different genetic markers for ER stress (*PERK*, *eiF2*, *CHOP*, *PTP1b*, *BIP*, *IRE1*, *ATF6*, *XBP1*, and *124-3p miRNA*) were quantified by qRT-PCR. (**J**) A representative image of the endoplasmic reticulum after staining with ER Tracker (green) in nontreated cells (CTRL), cells treated with tunicamycin (ER_s), and tunicamycin + EVs (EVs). Nuclei were stained with DAPI (blue). Bar 10 µm. Values expressed as mean ± SD. * *p* < 0.05, ^###^ or *** *p* < 0.001. Statistical significance indicated as asterisks (*) when comparing the EVs group to ER_s group and as number signs (#) when comparing ER_s group to control.

ER stress is recognized as an important factor in the pathogenesis of corneal injury, affecting resident corneal stem cells and impairing their metabolic homeostasis and regenerative potential. Therefore, we tested the efficacy of EVs treatment to reverse ER stress in CSSCs. Tunicamycin induced sustained ER stress and unfolded protein response (UPR) in CSSCs, demonstrated by the induction of ER-stress-associated genes (*PERK*, *eiF2*, *CHOP*, *PTP1b*, *BIP*, *IRE1*, *ATF6*, and *XBP1*) (Figure 9A–H). Most importantly, treatment with EVs reduced the gene expression of all these markers, compared to the tunicamycin-treated group (Figure 9A–H). Interestingly, compared to the ER stress condition, cells treated with EVs showed increased expression of the 124-3p miRNA, a potent ER stress suppressor, suggesting that EVs may stimulate the cellular intrinsic regulatory pathways for ER stress attenuation (Figure 9I). Moreover, these cells displayed a collapsed ER network and were characterized by the appearance of ER Tracker Green positive vesicular structures, which correspond to ER whorls that are associated with UPR and ER stress (Figure 9J). Interestingly, EVs treatment promoted ER net recovery, as demonstrated by the depletion of the sphere-like structures characteristic of ER whorls (Figure 9J). Altogether, our results show that EVs regulate the expression of multiple cellular factors to mitigate the ER stress in CSSCs.

### 3.6. EVs Promote Corneal Cells Metabolic Homeostasis under ER Stress Condition

**Figure 10:**
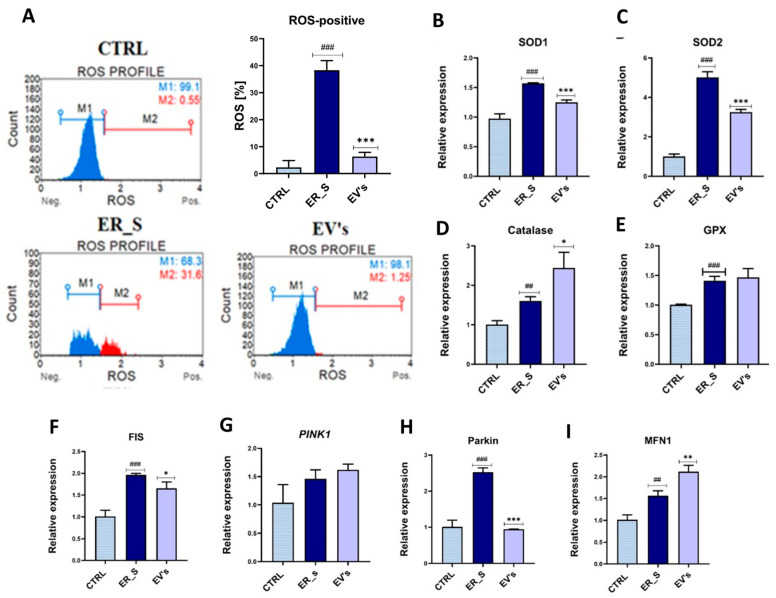
EVs alleviate ER-induced oxidative stress in CSSCs. (**A**) ROS accumulation in control (CTRL), ER_s, and EVs was tested using the Muse Analyzer Flow Cytometry. (**B**–**E**) The relative expression of genes encoding endogenous antioxidant enzymes (SOD1, SOD2, CAT, GPX) was tested using RT-qPCR. (**F**–**I**) The relative expression of genes related to the dynamics of mitochondria, i.e., FIS, MFN1, Parkin, and PINK1. All values expressed as mean ± SD. * *p* < 0.05, ^##^ or ** *p* < 0.01, ^###^ or *** *p* < 0.001. Statistical significance indicated as asterisks (*) when comparing the EVs group to ER_s group and as number signs (#) when comparing ER_s group to control.

Oxidative stress resulting from excessive ROS release during oxidative protein folding is intimately associated with ER chaperones and oxidoreductases overactivation, which lead to cell damage, the consequence of which is programmed cell death. To understand the mechanism by which EVs lower ER-stress-induced apoptosis, we analyzed the extent of oxidative stress. ER stress induced by tunicamycin increased the number of cells with detectable ROS accumulation (up to 37%), compared to the nontreated control (Figure 10A). Furthermore, the same cells exhibited increased relative expression of the main antioxidant enzymes transcripts (*SOD1*, *SOD2*, *CAT*, *GPX*), suggesting the establishment of a compensatory response aimed at overcoming the depleted antioxidant defenses (Figure 10B–E). Treatment of CSSCs with equine ASCs-derived EVs triggered a striking decline in the total ROS to less than 5% compared to untreated cells (Figure 10A). Moreover, cells treated with EVs showed a decrease in *SOD1* and *SOD2* expression compared to ER-stressed cells, suggesting an upstream modulation of oxidative stress, leading to attenuation of the engaged compensatory mechanisms (Figure 10B,C).

Mitochondrial biogenesis and dynamics alterations are known to play an important role in the development of oxidative stress and overall cellular metabolic imbalance. ER-stressed cells were characterized by the overexpression of the fis1/pink1/parkin pathway that promotes mitochondrial fission and mitophagy (Figure 10F–H), which can lead to increased oxidative stress in the cell. Interestingly, CSSCs treated with EVs decreased the expression of *Fis-1* and *Parkin* compared to ER-stressed cells (Figure 10F,H), while they exhibited higher expression of *MFN1* (Figure 10I). However, EVs did not reduce the gene expression level of PINK1 in CSSCs with ER stress (Figure 10G).

**Figure 11:**
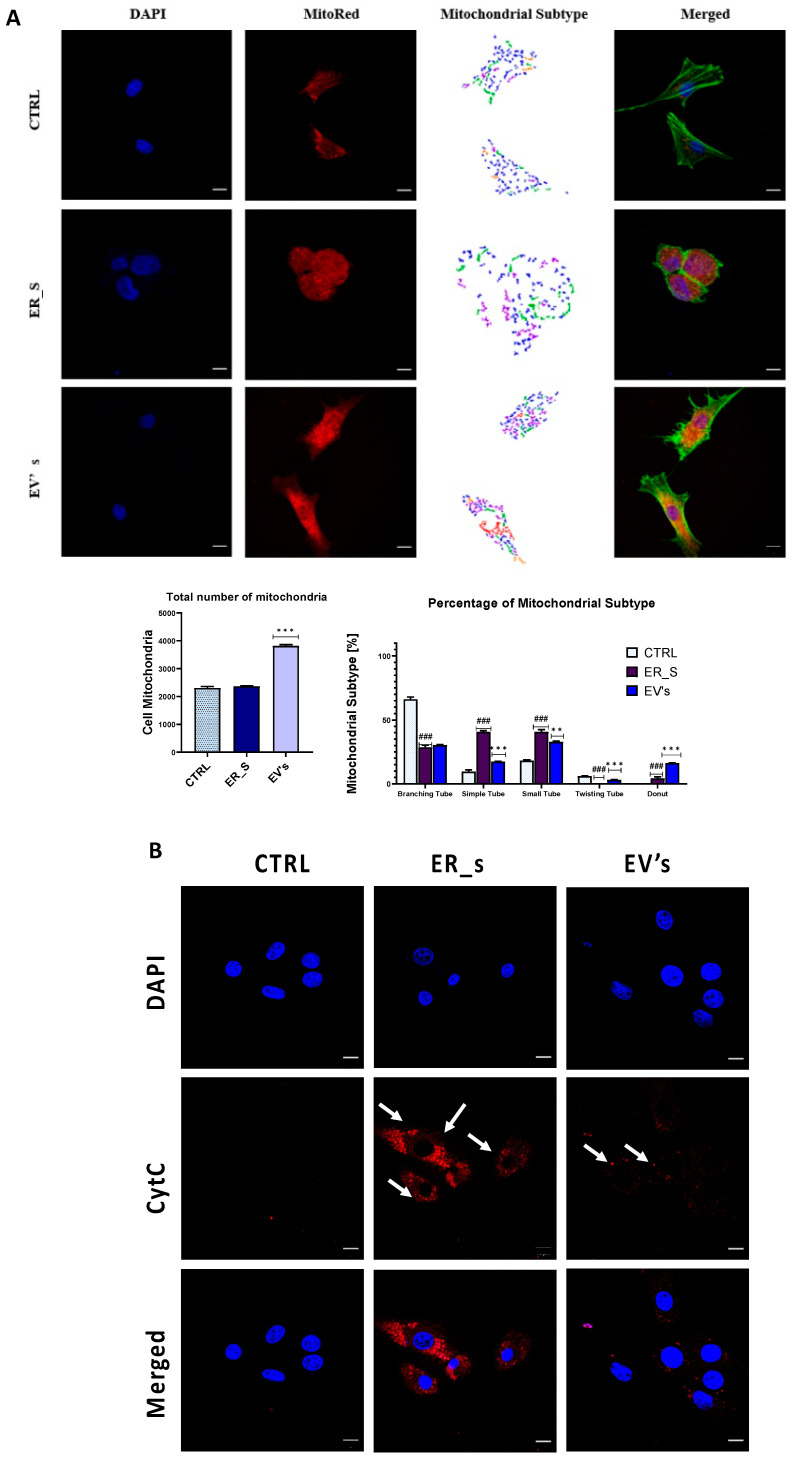
EVs protect mitochondria from ER-induced stress in CSSCs. (**A**) Representative mitochondrial staining with MitoRed (red), where the nuclei (DAPI, blue) and the F-actin (phalloidin, green) were stained. The morphology, number, and shape of mitochondria in the healthy control, ER_s, and EVs treatment groups were quantified and represented in graphs. (**B**) Cytochrome C immunostaining was visualized with confocal microscopy. Nuclei were stained with DAPI (blue) and cytochrome C with specific antibodies (red). Arrows indicate cytochrome C signal; bar 10 µm. All values expressed as mean ± SD. ** *p* < 0.01, ^###^ or *** *p* < 0.001. Statistical significance indicated as asterisks (*) when comparing the EVs group to ER_s group and as number signs (#) when comparing ER_s group to control.

The number and type of mitochondria in the experimental cells were also analyzed using the Micro P program. Cells treated with EVs contained the highest number of mitochondria (Figure 11A). Interestingly, in most cases, the detected types of mitochondria were comparable to the healthy control group, with the exception of the “Branching tube” and “donut” types (Figure 11A). Excessive Cytochrome C (Cyt C) release is a prominent hallmark of mitochondrial dysfunction, known to irreversibly initiate proapoptotic pathway when translocated into the cytoplasm. As shown in Figure 11B, tunicamycin caused mitochondrial Cyt C release in the CSSC cytosol, as evidenced by the increased Cyt C positive fluorescent signal, in contrast to healthy cells, which were characterized by a negative staining signal (Figure 11B). Importantly, addition of EVs to ER-stressed cells led to reduced levels of cytosolic Cyt C (Figure 11B). These results indicate that EVs are able to relieve the damaging oxidative stress triggered by the ER stress, mainly through the enhancement of mitochondrial integrity.

## 4. Discussion

Corneal injuries in horses are a very common ophthalmologic presentation that requires intensive medical or surgical treatment. These injuries are characterized by significant inflammatory and apoptotic responses, as well as intense oxidative and ER stress, which collectively increase the risk of fibrosis and scar formation [83]. These changes can alter the corneal shape and transparency, leading to visual impairment. Moreover, severe corneal injuries may contribute to the development of other complications, including corneal ulcers [84], corneal melting, and even vision loss [85]. Currently, there are no approved therapies that would reduce apoptosis and inflammation, and at the same time also improve CSSCs survival and regenerative capacity through mitigation of ER stress. In the present study, we demonstrated that EVs isolated from healthy equine ASCs improved the metabolic functions, stemness, survival, and proliferation potential of CSSCs following tunicamycin-induced ER stress.

Endoplasmic reticulum is a crucial cellular organelle that regulates soluble and membrane proteins’ turnover. Disrupted ER homeostasis, proteosynthesis overload, and secretory pathway failure have been described as critical modulators of stem cells dysfunction [86]. ER stress manifests primarily through the activation of stress-sensing transmembrane proteins that change the balance between anti- and proapoptotic markers towards programmed cell death [86]. In this study, the exposure of CSSCs to tunicamycin, a nucleoside antibiotic known to elicit a rapid and robust ER stress response, resulted in the immediate activation of the apoptotic pathway, and reduced cell survival rate, as a consequence of upregulation of key apoptosis regulators including p53, p21, BAX, and Caspase 3. Consequently, stressed cells displayed reduced proliferative and migratory capacities, and a disrupted cell cycle, being also associated with the attenuation of CSSCs’ proliferative, self-renewal and repair potential. Most importantly, treatment with EVs to ER-stressed cells countered the tunicamycin-damaging effects and partially restored the control phenotype in all these parameters. Our data are in agreement with studies showing the beneficial effects of EVs in treating corneal injuries, such as that of Samaeekia and colleagues [40] showing that CSSC-derived EVs promote corneal epithelial cells survival, proliferative potential, migration, and improvement in in vivo epithelial wound closure in a mouse model.

Inflammatory regulators play a significant role in the regulation of cell death, immune response, and fibrosis in the cornea, and dysregulation of these molecules can result in a variety of negative outcomes, including visual impairment [56,87,88,89,90,91,92,93,94,95,96,97,98]. Shojaati and colleagues previously demonstrated that EVs reduce not only apoptosis, but also fibrosis and inflammation, of corneal tissue after injury [45]. A similar effect was demonstrated in the present study, as the CSSCs cultured under stress conditions exhibited significantly reduced expression of proinflammatory cytokines including IL-1α, IL-1β, IL-6, and TFNα, and the profibrotic mediator TGF-β, when treated with EVs. At the same time, EVs induced expression of the anti-inflammatory cytokines IL-4 and IL-10.

In response to the accumulation of aberrant synthetized and/or unfolded proteins resulting from ER stress, coactivation of proinflammatory pathways occurs [58]. All three ER stress sensors, IRE1, PERK, and ATF6, initiate activation of the NF-κB axis, leading to the induced expression of proinflammatory (IL-1β, TNF-α, IFN-β) and profibrotic scarring factors (TGF-β) [58,99]. Therefore, the observed anti-inflammatory response supports the use of EVs as anti-inflammatory agents protecting against a wide variety of inflammatory diseases, including tissue fibrosis [100]. The anti-inflammatory mechanism of EVs was recently explained by Harting and colleagues and by others who showed that MSCs-derived EVs reduce inflammatory reaction by inhibiting early neutrophil infiltration and inducing specific M2 macrophage phenotypes secreting anti-inflammatory cytokines (IL-4, IL-10) [40,46,47]. Furthermore, EVs derived from different sources have been characterized for their high content of potent immunoregulatory mediators, such as miR-146b, miR181, miR-223, and Y-RNA fragments that are known to potently mitigate inflammatory responses and suppress excess cytokines release [101,102,103].

Hence, a growing body of evidence implicates ER stress in the pathogenesis of inflammatory disorders including corneal injury. Here, for the first time, we demonstrate that EVs mitigate ER stress induced in CSSCs with tunicamycin. We show that EVs reduce the expression of *BIP* under the stress condition, which in turn activates *IRE1*, *PERK*, and *ATF6*—key regulators of ER stress, which is a critical step in the pathogenesis of corneal lesions [48]. Under molecular stress conditions, these markers are activated and initiate UPR response, playing an important role in fibrosis and in scar tissue formation, both of which are crucial for corneal injury healing [58]. Previous investigations similarly demonstrated the potential of EVs in regulating ER stress events. Zhang and colleagues [104] found that human MSCs-derived EVs protect cardiac cells against hyperactive ER stress induced by hypoxia/reoxygenation (H/R) injury, through the downregulation of ER stress markers including *GRP78*, *CHOP*, *IRE1α*, and *ATF6*. Likewise, Bitirim and collaborators [105] demonstrated the efficacy of EVs in attenuating ER stress in hyperglycemic cardiomyocytes, thus confirming the great potential of EVs in countering the cellular distress associated with improper folding proteins.

Upon accumulation of misfolded proteins derived from ER stress, a cluster of chaperone systems are activated, including the UPR, in order to alleviate the perceived microenvironment discrepancy [106]. These processes are correlated with increased ROS production and depleted antioxidant defenses. The ensuing oxidative stress further initiates severe mitochondrial dysfunction that impairs cellular metabolic homeostasis and contributes to programmed cell death [107]. In the present investigation, tunicamycin triggered excessive ROS production, disrupted antioxidant balance, and caused profound mitochondrial failure evidenced by the increased release of cytochrome C, and an increase in fission over fusion. However, the addition of isolated ASCs-derived EVs to injured CSSCs lowered the extent of the oxidative stress, ameliorated the overall mitochondrial dynamics, and restored their integrity both by regulating *PINK1*, *MFF*, *MFN*, and *PARKIN* transcripts expression, and by reducing cytochrome C leakage. Our data are in agreement with previous findings by Xin et al., who observed that EVs derived from ASCs significantly reduced ROS production in a model of UVA and UVB-induced skin damage. Furthermore, EVs have also been shown to alleviate high glucose and H_2_O_2_-induced overproduction of ROS in cardiomyocytes [105]. Finally, EVs have also been reported to rescue damaged mitochondria in injured and inflamed alveolar epithelial tissue through the reduction in mitochondrial reactive oxygen species (mtROS), restoration of normal levels of mtDNA replication, and improvement of oxidative phosphorylation and overall biogenesis [108]. The researchers further suggested that MSCs-derived EVs were able to transfer functional mitochondria to injured cells, which subsequently promoted mitochondrial network recovery [108].

The relevance of this research work spans beyond the development of novel therapeutic approaches to treat corneal injuries in horses, being also of great interest for the application in humans. In this regard, our results complement previous studies demonstrating the beneficial role of EVs in treating human corneal injuries [64,109,110], providing valuable insights for the advancement of this field.

Our results are based on a comprehensive characterization of the effect of MSCs-derived EVs in vitro; therefore, they are limited to a specific experimental model with horse-derived CSSCs. However, the strong evidence showing that MSCs-derived EVs ameliorate the ER stress in CSSCs is promising regarding their application in a clinical setting. Further research is required to develop the optimal conditions for the clinical application of MSCs-derived EVs in vivo, focused on different aspects such as a more detailed analysis of the effect of the EVs in other relevant cellular pathways, how EVs ameliorate other type of cellular stresses associated with corneal injuries, which are the optimal conditions for culture of MSCs and isolation of EVs, or the clinical implementation of the treatment in horses with corneal injuries. Collectively, our data and previous findings clearly demonstrate the efficacy of MSCs-derived EVs in palliating ER-stress-associated cellular stress, including oxidative stress and mitochondrial failure, most likely through the transfer of functional mitochondria and other antioxidant mediators.

## 5. Conclusions

Our findings demonstrate that MSCs-derived EVs represent an innovative and promising therapeutic strategy for the transfer of bioactive mediators which regulate various cellular and molecular signaling pathways. It is, thus, reasonable to conclude that mitigation of ER stress might represent an efficacious approach for the modulation of inflammation, fibrosis, and scarring following corneal injury. Altogether, our study paves the road for the future development of ground-breaking targeted therapies for corneal healing in horses.

## Figures and Tables

**Table 1:** List of antibodies used for immunofluorescence.

Antibodies	Concentrations	CAT Numbers	Company
**KI-67**	1:1000	orb10033	Biorbyt (Janki, Poland)
**IL-1β**	1:500	ab9722	Abcam (Cambridge, UK)
**Cas3**	1:1000	c8487	Sigma (Poznan, Poland)
**CytC**	1:500	Nb100-56503	Novous (Poznan, Poland)

IL-1β—Interleukin 1β; Cas3: Caspase 3; CytC: Cytochrome C.

**Table 2:** Sequences of primers used in qPCR.

Gene	Primer Sequence (5′->3′)
***BAX***	F: GGCACCTCTTCCCTCCTTTCT
	R: CGATGCGCTTGAGACACTCG
***BCL2***	F: TTCTTTGAGTTCGGTGGGGT
	R: GGGCCGTACAGTTCCACAA
***p21***	F: GAAGAGAAACCCCCAGCTCC
	R: TGACTGCATCAAACCCCACA
***p53***	F: TACTCCCCTGCCCTCAACAA
	R: AGGAATCAGGGCCTTGAGGA
***Cas3***	F: GGCAGACTTCCTGTATGCGT
	R: CCATGGCTACCTTGCGGTTA
***Cas6***	F: GCCTACAGAAGAAGTTTGGGG
	R: AGGGTTAGGTGCCAGAAGAA
***Cas8***	F: ACTGTGATGTTGCTGGGACT
	R: CTTTCTCCTGGTGCATCTATCG
***Cas9***	F: CACCTTCCCAGGCTTTGTCT
	R: GGCTCTGGCCTCAGTAAGTT
***IL-1α***	F: CCAAGCTGAACTTCAAGGAGAGCGT
	R: CTCAGCACATGCTCAGCAAGTGAC
***IL-1β***	F: TATGTGTGTGATGCAGCTGTG
	R: ACTCAAATTCCACGTTGCCC
***IL-6***	F: CGTCACTCCAGTTGCCTTCT
	R: GCCAGTACCTCCTTGCTGTT
***TNF α***	F: TCCTACCCGTCCAAGGTCAA
	R: CTCATACCAGGGCTTGGCTT
***IL-4***	F: CGTCACTCCAGTTGCCTTCT
	R: GCCAGTACCTCCTTGCTGTT
***IL-10***	F: CTAGGGAACGAAGCATCCAGG
	R: TCAGGAGAGAGGTACCACAGG
***TGF β***	F: ATTCCTGGCGCTACCTCAGT
	R: GCTGGAACTGAACCCGTTGAT
***PERK***	F: GTGACTGCAATGGACCAGGA
	R: TCACGTGCTCACGAGGATATT
***eIF2***	F: AGTCTTCAGGCATTGGCTCC
	R: CCGAGTGGGACATGTATCGG
***CHOP***	F: AGCCAAAATCAGAGCCGGAA
	R: GGGGTCAAGAGTGGTGAAGG
***ATF6***	F: CAGGGTGCACTAGAACAGGG
	R: AATGTGTCTCCCCTTCTGCG
***PTP1B***	F: TGGAAGGAGCTCTCCCATGA
	R: GGAAGGGCTTCCAGTGAGTC
***BIP***	F: CTGTAGCGTATGGTGCTGCT
	R: CATGACACCTCCCACGGTTT
***IRE1***	F: GAATCAGACGAGCACCCGAA
	R: TTTCTTGCAGAGGCCGAAGT
***XBP1***	F: TTACGCGAGAAAACTCATGGCC
	R: GGGTCCAAGTTGAACAGAATGC
***SOD1***	F: CATTCCATCATTGGCCGCAC
	R: GAGCGATCCCAATCACACCA
***SOD2***	F: GGACAAACCTGAGCCCCAAT
	R: TTGGACACCAGCCGATACAG
***Cat***	F: ACCAAGGTTTGGCCTCACAA
	R: TTGGGTCAAAGGCCAACTGT
***GPX***	F: TCGAGCCCAACTTCACACTC
	R: AAGTTCCAGGCGACATCGTT
***FIS***	F: GGTGCGAAGCAAGTACAACG
	R: GTTGCCCACAGCCAGATAGA
***MNF1***	F: AAGTGGCATTTTTCGGCAGG
	R: TCCATATGAAGGGCATGGGC
***Parkin***	F: TCCCAGTGGAGGTCGATTCT
	R: CCCTCCAGGTGTGTTCGTTT
***PINK1***	F: GCACAATGAGCCAGGAGCTA
	R: GGGGTATTCACGCGAAGGTA
***CD63***	F: GTTCTTCTGCTGGCCTTCTG
	R: CTGCATCCTGTCCACAATCG
***CD9***	F: CCGATTCGACTCTCAGACCA
	R: CTGTTCTACTCCACCAGCCA
***CD81***	F: CACAGACCACCAACCTTCTC
	R: CAGGCAAAGAGGATGACGAG
***GAPDH***	F: GATGCCCCAATGTTTGTGA
	R: AAGCAGGGATGATGTTCTGG

*BAX*: BCL-2-associated X protein; *BCL-2*: B-cell lymphoma 2; *p21*: cyclin-dependent kinase inhibitor 1A; *p53*: tumor suppressor p53; *Cas3*: Caspase 3; *Cas6*: Caspase 6; *Cas8*: Caspase 8; *Cas9*: Caspase 9; *IL-1α*: Interleukin 1α *IL-1β:* Interleukin1β; *IL-4:* Interleukin 4: *IL-6*: Interleukin 6; *IL-10*: Interleukin 10; *TNFα*: Tumor necrosis factor; *TGFβ*: Transforming growth factor beta; *PERK*: Protein Kinase RNA-Like ER Kinase; *eIF2-α*: Eukaryotic Initiation Factor 2 α; *CHOP*: C/EBP homologous protein; *ATF6*: Activating Transcription Factor 6; *IRE1*: Inositol-requiring enzyme 1; *XBP1*: X-box binding protein; *PTP1b*: Protein Tyrosine Phosphatase 1B; *BIP*: Binding immunoglobulin protein; *SOD1*: Superoxide dismutase [Cu-Zn]; *SOD2*: Superoxide dismutase [Mn]; Cat: Catalase; *GPX*: Glutathione peroxidase; *FIS1*: Mitochondrial fission 1 protein; *MFN1*: Mitofusin-1; *Parkin*: E3 ubiquitin ligase parkin; *PINK1*: PTEN Induced Kinase 1; *CD63*, *CD81*, *CD9*: Clusters of differentiation; *GAPDH*: Glyceraldehyde 3-phosphate dehydrogenase.

**Table 3:** Sequences of microRNA primers used in qPCR.

Primer miRNAs	Primer Sequence (5′->3′)
**miR155**	TTAATGCTAATCGTGATAGGGGTT
**miR146a-5p**	TGAGAACTGAATTCCATGGGTT
**miR124-3p**	TAAGGCACGCGGTGAATGCCAA
**miR17-5p**	CAAAGTGCTTACAGTGCAGGTAG
**miR101-1/2**	TACAGTACTGTGATAACTGAA

## Data Availability

The datasets used and/or analyzed during the current study are available from the corresponding author on reasonable request.
